# Prevalence and Pattern of Cognitive Impairment in Patients on Maintenance Hemodialysis

**DOI:** 10.7759/cureus.84649

**Published:** 2025-05-22

**Authors:** Jehangir Afzal Mobushar, Anjum Shahzad, Bushra Mumtaz, Iqra Naeem, Adnan Ahmad Zafar, Muhammad Irfan Jamil, Syed Safiullah Shah, Muhammad Awais, Adeel Ahmed

**Affiliations:** 1 Medicine, Rashid Hospital, Dubai, ARE; 2 Internal Medicine, Services Hospital Lahore, Lahore, PAK; 3 Nephrology, Lahore General Hospital, Lahore, PAK; 4 Cardiology, Punjab Institute of Cardiology, Lahore, PAK; 5 Psychiatry and Behavioral Sciences, Lahore General Hospital, Lahore, PAK; 6 Nephrology, Islam Medical College, Sialkot, PAK; 7 Medicine, Lahore General Hospital, Lahore, Lahore, PAK

**Keywords:** cognitive assessment, hd ( hemodialysis ), memory decline, montreal cognitive assessment (moca), socio-demographic

## Abstract

Background

Despite the rising burden of end-stage renal disease (ESRD), cognitive assessment is not routinely incorporated into dialysis care, particularly in low-resource settings. This study aimed to assess the prevalence, severity, and predictors of cognitive impairment among patients on maintenance hemodialysis.

Methods

A descriptive cross-sectional study was conducted at a tertiary dialysis center from December 2023 to May 2024. Patients aged ≥18 years undergoing hemodialysis for six months or more were enrolled. Exclusion criteria included neurological disorders, severe psychiatric illness, or medications affecting cognition. Cognitive function was evaluated using the Urdu-validated Montreal Cognitive Assessment (MoCA), with scores <26 indicating impairment. Domain-wise deficits were classified using 1.5 standard deviation below the normative mean. Statistical analysis included chi-square tests, t-tests, and multivariable logistic regression.

Results

Out of 198 hemodialysis patients, 116 (58.6%) exhibited cognitive impairment. Severity was categorized as mild in 79 (39.9%), moderate in 31 (15.7%), and severe in eight (4.0%) patients. Multidomain impairment was present in 108 (54.5%), while 18 (9.1%) had single-domain and 72 (36.4%) had no impairment. Older age (p<0.001), lower education (p<0.001), low socioeconomic status (SES) (p=0.045), and longer dialysis duration (p<0.001) were significantly associated with cognitive impairment. Biochemical predictors included lower hemoglobin and albumin (p=0.018 and p=0.034), and higher phosphate and intact parathyroid hormone (iPTH) (p=0.001 and p=0.042). On regression analysis, age (adjusted odds ratio (AOR)=1.088, 95% confidence interval (CI)=1.031-1.149), education ≤12 years (AOR=10.423, 95% CI=1.199-90.633), low SES (AOR=9.075, 95% CI=1.473-55.916), dialysis duration (AOR=1.047, 95% CI=1.010-1.085), and biochemical markers remained significant.

Conclusion

Cognitive impairment, particularly multidomain, is highly prevalent among hemodialysis patients and frequently unrecognized. Integration of cognitive screening into routine nephrology care is essential to enable early intervention and improve long-term patient outcomes.

## Introduction

End-stage renal disease (ESRD) remains a major global health burden, affecting more than 850 million individuals and leading to significant morbidity and mortality due to its systemic complications [1]. Hemodialysis (HD), a cornerstone of renal replacement therapy, though lifesaving, is associated with neurocognitive complications due to repetitive intradialytic hemodynamic instability and chronic uremia. Mounting evidence suggests that patients receiving HD are particularly vulnerable to neurological impairment including cognitive dysfunction, which is often underdiagnosed in clinical nephrology practice [2,3]. While earlier views considered cognitive issues as incidental or age-related, current literature supports a more intrinsic association between ESRD pathophysiology and cerebral dysfunction, including small-vessel ischemia, oxidative stress, and white matter lesions [4].

Cognitive impairment (CI) in ESRD refers to dysfunction in areas such as attention, executive functioning, memory, language, and visuospatial processing, often presenting subtly but progressing over time [5]. Among individuals undergoing maintenance hemodialysis, CI prevalence estimates range from 40% to 80%, with the most affected domains being executive function and attention [2,6]. These impairments are attributed to multifactorial processes, including cerebral hypoperfusion during HD, uremic neurotoxicity, anemia, hyperphosphatemia, and chronic inflammation [1,4]. Moreover, impaired cognition adversely affects patients' ability to adhere to treatment, comprehend medical advice, and maintain dialysis schedules, thereby increasing hospitalizations and mortality [7]. Hemodialysis patients with CI have been shown to experience increased functional dependency compared to the general (non-dialysis) population, along with reduced ability to engage in activities of daily living [3]. In contrast, higher educational attainment, better dialysis adequacy (Kt/V), and engagement in physical activity have been associated with protective effects on cognitive function [8-10]. Moreover, CI has been found to significantly compromise health-related quality of life, especially among patients with multi-domain cognitive involvement, who frequently report diminished psychosocial satisfaction, reduced autonomy, and poorer mental health outcomes [2,3].

Despite a high prevalence of cognitive impairment in the hemodialysis population, routine assessment of cognitive status is not standard practice in most dialysis centers, including in Pakistan. In clinical routines, nephrologists often focus on biochemical parameters, fluid balance, and dialysis efficacy, inadvertently overlooking subtle cognitive symptoms that may compromise patient outcomes. Furthermore, most existing studies have been conducted in high-income countries with limited data available from low- and middle-income regions, where the burden of ESRD is rapidly increasing [1,2,10,11]. Local studies are often constrained by small sample sizes, non-standardized tools, or lack of domain-specific analysis. Without early recognition and intervention, patients with CI may be at higher risk for non-compliance, psychological distress, and treatment failure. Therefore, there is a compelling need to generate locally relevant data on cognitive patterns in HD patients to inform clinical care, design targeted screening, and support multidisciplinary intervention models.

## Materials and methods

This descriptive cross-sectional study was conducted at the Dialysis Center, Department of Nephrology, Lahore General Hospital, Lahore, over a six-month period from December 2023 to May 2024. Ethical approval was granted by the Institutional Review Board (IRB # 202/12/2023), and written informed consent was obtained from all the participants prior to inclusion in the study.

Individuals aged 18 years or older receiving maintenance hemodialysis twice or thrice weekly for at least six months were eligible for inclusion, provided they were able to comprehend the local language and complete cognitive assessments independently or with minimal assistance. Participants were excluded if they had a documented diagnosis of any neurodegenerative disease such as Alzheimer’s or Parkinson’s, advanced dementia, major psychiatric illness, history of stroke, traumatic brain injury, central nervous system infection, or severe hepatic dysfunction. Additional exclusion criteria included significant visual or auditory impairment affecting cognitive testing, previous renal transplantation with a return to dialysis, and current use of medications known to impair cognition, including but not limited to benzodiazepines, antipsychotics, and corticosteroids taken within the preceding 30 days.

Data were collected through structured face-to-face interviews and a review of medical records. Demographic information, including age, gender, education level, and marital status, along with clinical details such as comorbidities, dialysis duration, and recent laboratory values, were recorded using a standardized data collection form. Cognitive assessments were performed by trained medical personnel using the Urdu-validated version of the Montreal Cognitive Assessment (MoCA) [12]. To minimize dialysis-related fatigue or hemodynamic variability, cognitive testing was conducted on the long interdialytic interval, prior to the dialysis session, in a quiet and distraction-free setting.

Cognitive function was assessed using the MoCA, a 30-point screening tool covering seven cognitive domains: visuospatial/executive (5 points), naming (3 points), attention (6 points), language (3 points), abstraction (2 points), delayed recall (5 points), and orientation (6 points). A total score below 26 was considered indicative of cognitive impairment. One additional point was added for participants with 12 or fewer years of education, as per standard MoCA guidelines. Severity was categorized as mild (MoCA 18-25), moderate (10-17), and severe (<10). The pattern of cognitive impairment will be assessed by analyzing individual domain scores within the MoCA. In accordance with standard cognitive research methodology, domain-wise impairment was defined using the criterion of 1.5 standard deviations below the mean scores derived from the cognitively preserved group; thus, a domain was labeled impaired if the patient scored ≤4 in Visuospatial (cut-off: 4.57), ≤1 in Naming (cut-off: 1.67), ≤4 in Attention (cut-off: 4.69), ≤1 in Language (cut-off: 1.78), ≤1 in Abstraction (cut-off: 1.70), ≤3 in Delayed Recall (cut-off: 3.70), and ≤4 in Orientation (cut-off: 4.63) [3,4].

Data were analyzed using IBM SPSS Statistics version 26.0 (IBM Corp, Armonk, NY). Descriptive statistics were used to summarize demographic and clinical characteristics. Continuous variables were reported as mean ± standard deviation, while categorical variables were presented as frequencies and percentages. The prevalence of cognitive impairment was determined based on MoCA scores, and the pattern was assessed by evaluating domain-wise performance. Between-group comparisons were made using the independent samples t-test for continuous variables and chi-square test for categorical variables. Univariable logistic regression was performed to explore the association between cognitive impairment and individual predictors. Variables with p≤0.20 or clinical relevance were included in multivariable logistic regression to identify independent predictors. Odds ratios with 95% confidence intervals were reported, and a p-value≤0.05 was considered statistically significant.

## Results

In this study of 198 hemodialysis patients, cognitive impairment (MoCA <26) was observed in 116 patients (58.6%). The severity distribution of cognitive function among the 198 hemodialysis patients revealed that 82 (41.4%) had normal cognition, 77 (38.9%) had mild impairment, 31 (15.7%) exhibited moderate impairment, and eight (4.0%) had severe cognitive impairment. Regarding the pattern of cognitive deficits, 72 (36.4%) patients showed no impairment, 18 (9.1%) had single-domain impairment, while multidomain impairment was observed in 108 (54.5%) individuals. Impairment was significantly more frequent in patients aged 41-70 years (93, 80.2%; odds ratio (OR) = 10.37, 95% confidence interval (CI): 5.34-20.14; p<0.001) and those with ≤12 years of education (113, 68.9%; p<0.001). Low socioeconomic status was associated with higher impairment (38, 73.1%; p=0.045). Diabetes (p<0.001), hypertension (p<0.001), and cardiac disease (p=0.009) showed significant associations. Impaired patients had lower hemoglobin and albumin, and higher phosphate and intact parathyroid hormone (iPTH) levels (all p<0.001) (Table 1).

**Table 1:** Association Between Demographic, Clinical, and Laboratory Parameters and Cognitive Impairment in Hemodialysis Patients. Categorical variables were analyzed using chi-square (χ²) test, and results are reported as frequencies (*n*), percentages (%), odds ratios (OR), 95% confidence intervals (CI), and corresponding p-values. Continuous variables were compared using independent sample t-tests, and results include means ± standard deviations (SD), mean differences with 95% CI, and p-values. Abbreviations: OR: odds ratio; CI: confidence interval; SD: standard deviation; MoCA: Montreal Cognitive Assessment; AV fistula: arteriovenous fistula, CVC: central venous catheter; iPTH: intact parathyroid hormone; g/dL: grams per deciliter; mg/dL: milligrams per deciliter; pg/mL: picograms per milliliter.

Variable	Subgroup	Cognitive Impairment Present *n (%)*	Cognitive Impairment Absent *n (%)*	Test Statistic *χ² / t*-value	Effect Size (OR or Mean Difference) 95% CI	p-value
Age Group	18–40 years	23 (28.0%)	59 (72.0%)	χ²=53.79	OR=10.37 (5.34–20.14)	<0.001
	41–70 years (Ref)	93 (80.2%)	23 (19.8%)			
Gender	Male	59 (55.7%)	47 (44.3%)	χ²=0.805	OR=1.30 (0.73–2.29)	0.370
	Female	57 (62.0%)	35 (38.0%)			
Marital Status	Married	67 (64.4%)	37 (35.6%)	χ²=5.85	-	0.054
	Single	28 (45.9%)	33 (54.1%)			
	Widow/Divorced (Ref)	21 (63.6%)	12 (36.4%)			
Education Level	≤12 years	113 (68.9%)	51 (31.1%)	χ²=41.90	OR=0.044 (0.013–0.149)	<0.001
	>12 years	3 (8.8%)	31 (91.2%)			
Socioeconomic Status	Low	38 (73.1%)	14 (26.9%)	χ²=6.19	-	0.045
	Middle	51 (52.6%)	46 (47.4%)			
	High	27 (55.1%)	22 (44.9%)			
Dialysis Frequency	Twice	93 (55.4%)	75 (44.6%)	χ²=4.76	OR=2.65 (1.08–6.51)	0.029
	Thrice	23 (76.7%)	7 (23.3%)			
Comorbidity – Diabetes	Yes	54 (83.1%)	11 (16.9%)	χ²=23.92	OR=5.62 (2.70–11.69)	<0.001
	No	62 (46.6%)	71 (53.4%)			
Comorbidity – Hypertension	Yes	63 (78.8%)	17 (21.2%)	χ²=22.50	OR=4.54 (2.38–8.68)	<0.001
	No	53 (44.9%)	65 (55.1%)			
Comorbidity – Autoimmune Disease	Yes	10 (43.5%)	13 (56.5%)	χ²=2.45	OR=0.50 (0.21–1.20)	0.118
	No	106 (60.6%)	69 (39.4%)			
Comorbidity – Cardiac Disease	Yes	41 (73.2%)	15 (26.8%)	χ²=6.89	OR=2.44 (1.24–4.81)	0.009
	No	75 (52.8%)	67 (47.2%)			
Vascular Access	AV Fistula	86 (55.5%)	69 (44.5%)	χ²=2.83	OR=1.85 (0.90–3.82)	0.092
	CVC	30 (69.8%)	13 (30.2%)			
Age	-	Mean±SD	Mean±SD	t-test	-	<0.001
(Continuous)	-	47.63±9.86	34.54±10.99	t=-8.61	-13.09 (–16.04 to –10.09)	
Dialysis Duration (Months)	-	38.22 ± 20.98	24.85±14.46	t=-5.31	-13.37 (–18.34 to –8.40)	<0.001
Hemoglobin (g/dL)	-	9.30±1.92	10.90±2.09	t=5.48	+1.60 (1.02 to 2.18)	<0.001
Corrected Serum Calcium (mg/dL)	-	7.87±0.92	8.41±0.87	t=4.21	+0.54 (0.29 to 0.80)	<0.001
Serum Phosphate (mg/dL)	-	5.82±0.97	5.12±1.12	t=-4.62	-0.71 (–1.01 to –0.41)	<0.001
Serum Uric Acid (mg/dL)	-	7.01±1.72	6.57±1.75	t=-1.73	-0.44 (–0.93 to 0.06)	0.085
Serum iPTH (pg/mL)	-	401.52±185.54	244.43±158.66	t=-6.40	-157.19 (–206.96 to –108.72)	<0.001
Serum Albumin (g/dL)	-	3.34±0.58	3.84±0.50	t=6.43	+0.50 (0.34 to 0.65)	<0.001

Cognitive impairment was significantly associated with deficits across all MoCA domains. The most affected were visuospatial function 96 (48.5%), abstraction 92 (46.5%), delayed recall 80 (40.4%), and attention 69 (34.8%), all with p<0.001. Mean total MoCA score was lower in impaired individuals (18.60±4.69 vs. 27.20±1.48; p<0.001) (Table 2).

**Table 2:** Association of Individual MoCA Domains (Categorical and Continuous) With Cognitive Impairment Among Hemodialysis Patients (n = 198). MoCA impairment cut-offs for categorical domains were based on established norms. Chi-square test was used for categorical variables and independent samples t-test for continuous variables. All p-values are two-tailed. Reference group: “Not Impaired” (categorical) and “No Cognitive Impairment” (MoCA ≥26). MoCA: Montreal Cognitive Assessment

MoCA Domain	Impaired n (%)	Not Impaired n (%)	Test Value	p-value	Mean (±SD) Impaired	Mean (±SD) Not Impaired	Mean Difference	t-value	p-value
Visuospatial (Max 5)	96 (48.5%)	102 (51.5%)	χ²=84.05	<0.001	3.70±1.07	4.90±0.30	1.204	11.47	<0.001
Naming (Max 3)	19 (9.6%)	179 (90.4%)	χ²=14.86	<0.001	2.01±0.64	2.40±0.49	0.394	4.88	<0.001
Attention (Max 6)	69 (34.8%)	129 (65.2%)	χ²=64.75	<0.001	4.06±1.42	5.40±0.54	1.342	9.29	<0.001
Language (Max 3)	8 (4.0%)	190 (96.0%)	χ²=5.89	0.015	2.18±0.54	2.51±0.50	0.331	4.44	<0.001
Abstraction (Max 2)	92 (46.5%)	106 (53.5%)	χ²=91.69	<0.001	1.21±0.50	1.94±0.24	0.732	13.63	<0.001
Delayed Recall (Max 5)	80 (40.4%)	118 (59.6%)	χ² = 94.89	<0.001	2.86±0.88	4.44±0.50	1.577	15.95	<0.001
Orientation (Max 6)	86 (43.4%)	112 (56.6%)	χ² = Not provided	NA	2.60±1.16	5.46±0.61	2.860	22.54	<0.001
Total MoCA Score (Max 30)					18.60±4.69	27.20±1.48	8.592	18.47	<0.001

Binary logistic regression revealed several independent predictors of cognitive impairment (MoCA <26) in hemodialysis patients. Significant associations were found for increasing age (adjusted odds ratio (AOR)=1.088, p=0.002), low education (AOR=10.423, p=0.034), low socioeconomic status (AOR)=9.075, p=0.017), longer dialysis duration (AOR=1.047, p=0.012), low hemoglobin (AOR= 0.702, p=0.018), high phosphate (AOR=3.180, p=0.001), elevated iPTH (AOR=1.004, p=0.042), and low albumin (AOR=0.297, p=0.034) (Table 3).

**Table 3:** Multivariate Logistic Regression Analysis of Factors Associated with Cognitive Impairment in Hemodialysis Patients. Multivariate binary logistic regression analysis of independent predictors of cognitive impairment (MoCA < 26) among hemodialysis patients (N=198). All categorical variables were coded using indicator contrast method with the last category as reference: Female for Gender, Widow/Divorced for Marital Status, High for Socioeconomic Status, >12 years for Education Level, Thrice for Dialysis Frequency, and "No" for all comorbidities. A p-value<0.05 was considered statistically significant. AOR: adjusted odds ratio; CI: confidence interval; SE: Standard Error; Wald: Wald chi-square statistic; AOR: adjusted odds ratio; CI: confidence interval; DM diabetes mellitus; HTN: hypertension; AVF: arteriovenous fistula; CVC: central venous catheter; iPTH: intact parathyroid hormone.

Variable	SE	Wald	p-value	AOR (95% CI)
Age	0.028	9.436	0.002	1.088 (1.031-1.149)
Gender (Male vs. Female)	0.705	3.460	0.063	0.270 (0.068-1.075)
Marital Status	-	0.569	0.752	-
Married vs. Widow/Divorced	0.805	0.339	0.561	0.626 (0.129-3.033)
Single vs. Widow/Divorced	0.927	0.556	0.456	0.501 (0.081-3.085)
Education Level (≤12y vs. >12y)	1.104	4.512	0.034	10.423 (1.199-90.633)
Socioeconomic Status	-	8.226	0.016	-
Low vs. High	0.928	5.651	0.017	9.075 (1.473-55.916)
Middle vs. High	0.820	0.124	0.725	1.334 (0.268-6.651)
Dialysis Duration (months)	0.018	3.323	0.012	1.047 (1.010-1.085)
Dialysis Frequency (Twice vs. Thrice)	1.020	0.644	0.422	0.441 (0.060-3.257)
DM (Yes vs. No)	0.705	1.532	0.272	0.418 (0.088-1.985)
HTN (Yes vs. No)	0.605	4.236	0.040	0.288 (0.088-0.943)
Autoimmune Disease (Yes vs. No)	1.031	0.221	0.638	0.616 (0.082-4.647)
Cardiac Disease (Yes vs. No)	0.793	0.422	0.516	0.597 (0.126-2.828)
Vascular Access (AVF vs. CVC)	0.769	1.008	0.315	0.462 (0.102-2.087)
Hemoglobin (g/dL)	0.150	5.577	0.018	0.702 (0.523-0.941)
Serum Calcium Corrected (mg/dL)	0.374	1.572	0.264	0.658 (0.316-1.370)
Serum Phosphate (mg/dL)	0.346	11.182	0.001	3.180 (1.614-6.265)
Serum Uric Acid (mg/dL)	0.190	0.006	0.937	0.985 (0.679-1.429)
Serum Fasting iPTH (pg/mL)	0.024	4.134	0.042	1.004 (1.000-1.008)
Serum Albumin (g/dL)	0.572	4.509	0.034	0.297 (0.097-0.911)

## Discussion

This cross-sectional study of 198 hemodialysis patients demonstrated a substantial burden of cognitive impairment, with 39.9% showing mild, 15.7% moderate, and 4.0% severe dysfunction. Multidomain impairment was identified in 54.5% of patients. These findings are consistent with Luo et al. (2020), who reported 80.91% overall impairment, including 37.19% mild and 43.72% major cognitive deficits [13]. Similarly, in the COGNITIVE-HD study observed that 71.1% had impairment in at least one domain, with 45.2% exhibiting multidomain involvement [3]. Another study from Germany reported 75% cognitive impairment, predominantly multidomain [14]. A study from Rome found 80% mild cognitive impairment, while a Chinese study reported 60.9% [10,15]. In contrast, some studies documented the comparably low prevalence of CI in HD patients from 23% to 31.5% [16,17]. Notably, many patients remain undiagnosed clinically, as Chan et al. (2022) observed limited subjective complaints [16]. Zhang et al. (2024) estimated a pooled 53% prevalence in hemodialysis populations [1]. Schorr et al. (2022) identified multidomain impairment early after dialysis initiation [4]. Differences across studies are attributable to variations in cognitive testing, age, education, dialysis duration, and criteria being used to assess cognitive functions. Routine cognitive screening remains crucial to identify unrecognized impairment and improve clinical outcomes.

In this study, a distinct pattern of cognitive deficits was observed among hemodialysis patients using the MoCA. The most commonly affected domains included visuospatial function (48.5%), abstraction (46.5%), memory (40.4%), attention (34.8%), and orientation, indicating widespread impairment across multiple cognitive areas. Delayed recall, executive functioning, and attention tasks were particularly challenging for many patients, consistent with prior findings that described prominent dysfunction in memory and executive domains among dialysis populations [10,13-15]. These deficits likely reflect vascular and uremic neuronal injury, rather than classic Alzheimer-type pathology, which typically impairs orientation and recognition earlier. Notably, naming ability and basic orientation were relatively preserved in the majority, suggesting selective subcortical vulnerability [18-20]. Schorr et al. (2022) similarly reported an early decline in processing speed and reasoning [4]. Such domain-specific impairments have clear clinical implications, especially in relation to dialysis self-care. As Chan et al. (2022) emphasized, executive dysfunction can hinder adherence to fluid, diet, and medication regimens [16]. Therefore, routine cognitive screening using tools like MoCA, which detect executive deficits missed by MMSE, should be integrated into dialysis care. Tailored interventions that accommodate attention and memory limitations may help support patient functioning.

In the present study, age was identified as a significant independent predictor of cognitive impairment in patients receiving maintenance hemodialysis. Individuals aged 41-70 years exhibited markedly higher impairment rates (93 of 116, 80.2%) compared to those aged 18-40 years (23 of 82, 28.0%), with an odds ratio of 10.37 (95% CI: 5.34-20.14; p<0.001). Each one-year increase in age was associated with an 8.8% higher likelihood of impairment (AOR=1.088; 95% CI: 1.031-1.149; p=0.002). These findings are supported by previous research reporting elevated odds for both mild (OR=1.09) and major cognitive impairment (OR=1.12) [13]. One study observed a markedly increased risk among individuals aged ≥75 years (OR=90.22) [17]. Similar associations have been described, indicating accelerated cerebral aging in dialysis populations, likely mediated by vascular injury, chronic uremia, and intradialytic hemodynamic fluctuations [5,14,21]. These results reinforce the importance of routine, age-adjusted cognitive screening and early intervention strategies in older adults on dialysis [22]. No significant association was observed between gender and cognitive impairment in this study (p=0.370), consistent with previous reports indicating no significant gender-based differences in cognitive status among hemodialysis patients [11,13]. Although a slight female predominance was noted in some studies, this association did not remain statistically significant after adjustment for confounders [2,15].

Educational attainment demonstrated a strong inverse association with cognitive impairment. Individuals with ≤12 years of education had significantly higher prevalence (113 of 164, 68.9%) compared to those with >12 years (3 of 34, 8.8%), with multivariate analysis confirming a tenfold increased risk (AOR=10.423; 95% CI: 1.199-90.633; p=0.034). These findings are consistent with earlier studies, which support the cognitive reserve hypothesis. Studies have consistently shown that patients on hemodialysis with lower educational attainment are at greater risk of cognitive impairment. Increasing age and limited formal education are frequently identified as important risk factors for reduced cognitive performance, especially in areas such as attention and memory. In contrast, language and naming abilities tend to remain relatively preserved in most patients [4,6,13].

Low socioeconomic status was also independently associated with cognitive impairment (AOR=9.075; 95% CI: 1.473-55.916; p=0.017). Prior studies in the general population have linked low SES with cognitive deficits through chronic stress, malnutrition, and reduced stimulation [23-25]. These findings emphasize the need to incorporate social factors into dialysis care.

Dialysis-related parameters, particularly duration and frequency, were significantly associated with cognitive impairment in this study. Patients with impairment had a longer mean dialysis duration (38.22±20.98 months) compared to those without impairment (24.85±14.46 months; p<0.001). In multivariate analysis, dialysis duration remained an independent predictor (AOR=1.047; 95% CI: 1.010-1.085; p=0.012), indicating that each additional month on dialysis increased the odds of impairment by 4.7%. This finding aligns with Luo et al. (2020), who identified dialysis vintage as an independent risk factor for both mild and major cognitive impairment (OR=1.02; p<0.001) [13]. Chen et al. (2023) observed a nearly fourfold increase in cognitive impairment risk after five years of dialysis (OR=3.99; p = 0.003), and Zeng et al. (2022) reported a negative correlation (β=-0.72; p<0.001) [11,17]. In contrast, Karakizlis et al. (2022) and Schorr et al. (2022) found no significant association, potentially due to shorter follow-up or different population characteristics [4,14]. Univariate analysis revealed higher cognitive impairment among patients undergoing thrice-weekly dialysis (23 of 30, 76.7%) compared to twice-weekly sessions (93 of 168, 55.4%; OR=2.65; p=0.029), though the association lost significance in multivariate regression, possibly due to smaller sample size. Zeng et al. (2022) similarly reported worse cognition with increased frequency (β=-7.36; p<0.001), while Schorr et al. (2022) found no significant relationship [4,11]. These results suggest that treatment-related cerebral stressors, rather than frequency alone, may contribute to cognitive decline. Vascular access type was not significantly associated with cognitive impairment (χ²=2.83; OR=1.85; 95% CI: 0.90-3.82; p=0.092), in line with findings by Drew et al. (2017), who reported no differences in cognitive decline trajectories based on access type [7]. These results indicate that access type may not be a primary determinant of cognitive outcomes in hemodialysis patients.

Diabetes mellitus was significantly associated with cognitive impairment on univariate analysis (χ²=23.92; OR=5.62; p<0.001); however, this association was not retained in the multivariate model. A similar pattern of attenuation was reported previously [13]. In contrast, some studies found diabetes to be independently linked with cognitive outcomes, including lower MoCA scores and increased subjective complaints [1,16]. Additionally, a higher frequency of diabetes was observed among cognitively impaired malnourished individuals (31.3% vs. 5.9%; p=0.034) [15]. Despite these inconsistencies, diabetes remains a clinically relevant and modifiable vascular risk factor in end-stage renal disease.

Hypertension was significantly associated with cognitive impairment on both univariate (χ²=22.50; OR=4.54; p<0.001) and multivariate analyses (AOR=0.288; 95% CI: 0.088-0.943; p=0.040), with absence of hypertension appearing protective. This may reflect unmeasured confounders such as cardiac dysfunction. Prior studies consistently support hypertension as a contributor to cognitive impairment [1,10,13,16].

In the present study, hemoglobin levels were significantly associated with cognitive impairment on both univariate (p<0.001) and multivariate analysis (AOR=0.702; 95% CI: 0.523-0.941; p=0.018). Cognitively impaired patients had approximately 1 g/dL lower hemoglobin compared to unimpaired individuals. Each 1 g/dL increase in hemoglobin corresponded to a 30% reduction in the odds of impairment. These results align with Karakizlis et al. (2022), who reported a positive association between hemoglobin and semantic fluency scores (β=0.63; p<0.01) [14], and with Pei et al. (2019), who found a modest correlation with MoCA scores [10]. Chen et al. (2023), however, did not observe a significant relationship, suggesting variability based on sample characteristics [17]. Anemia may contribute to cerebral hypoxia and impaired cognitive processing, emphasizing the importance of anemia correction in preserving cognitive function in dialysis patients.

Disordered mineral metabolism, particularly elevated serum phosphate and iPTH, was also associated with cognitive impairment. Higher phosphate levels increased the risk over threefold (AOR = 3.180; 95% CI: 1.614-6.265; p=0.001), while higher iPTH levels also independently predicted impairment (AOR=1.004; 95% CI: 1.000-1.008; p = 0.042). These results are consistent with Rotondi et al. (2023), who reported higher PTH in cognitively impaired patients (361 vs. 245 pg/mL; p=0.014), and Golenia et al. (2023), who linked PTH with neuropsychiatric symptoms [2,15]. Hyperphosphatemia and secondary hyperparathyroidism may contribute through vascular calcification, arterial stiffness, and inflammation, leading to cerebral injury. Corrected calcium was not independently associated, likely due to narrow distribution [26].

Serum albumin was significantly lower in cognitively impaired patients (3.34±0.58 vs. 3.84±0.50 g/dL; p<0.001), with multivariate analysis confirming a protective effect (AOR=0.297; 95% CI: 0.097-0.911; p=0.034). These results support prior findings linking hypoalbuminemia to cognitive dysfunction, likely due to malnutrition and chronic inflammation [3,10].

This study has several limitations. Being cross-sectional in design, it cannot establish causal relationships or capture changes in cognitive function over time. The use of MoCA, although sensitive to executive dysfunction, may not detect all subtle impairments compared to a full neuropsychological battery. Selection bias may be present due to single-center sampling and underrepresentation of older or more frail patients. Despite these limitations, the study highlights important clinical implications. Routine cognitive screening using accessible tools like MoCA can help identify patients at risk. Attention to modifiable factors such as anemia, mineral imbalance, malnutrition, and vascular risk may support cognitive health in the dialysis population.

## Conclusions

This study found that cognitive impairment is common among patients on maintenance hemodialysis, with many individuals showing difficulties in memory, attention, and executive function. Older age, lower education, longer dialysis duration, poor nutritional status, anemia, and disordered mineral metabolism were important contributing factors. These findings emphasize the need for regular cognitive screening in dialysis care. Identifying high-risk individuals early and addressing modifiable factors through targeted interventions may help preserve cognitive function and improve overall patient outcomes. Incorporating cognitive assessments into routine nephrology practice can play a key role in enhancing the quality of life for this vulnerable population.
